# Comparison of pregnenolone sulfate, pregnanolone and estradiol levels between patients with menstrually-related migraine and controls: an exploratory study

**DOI:** 10.1186/s10194-021-01231-9

**Published:** 2021-03-23

**Authors:** Cecilia Rustichelli, Elisa Bellei, Stefania Bergamini, Emanuela Monari, Flavia Lo Castro, Carlo Baraldi, Aldo Tomasi, Anna Ferrari

**Affiliations:** 1 Department of Life Sciences, University of Modena and Reggio Emilia, Modena, Italy; 2 Department of Surgery, Medicine, Dentistry and Morphological Sciences with Transplant Surgery, Oncology and Regenerative Medicine Relevance, University of Modena and Reggio Emilia, Modena, Italy; 3 School of Pharmacology and Clinical Toxicology, University of Modena and Reggio Emilia, Modena, Italy; 4 Department of Biomedical, Metabolic and Neural Sciences, Unit of Medical Toxicology, Headache Centre and Drug Abuse; University of Modena and Reggio Emilia, Via del Pozzo, 71, 41124 Modena, Italy

**Keywords:** Pregnenolone sulfate, Pregnanolone, Estradiol, Migraine, Menstrually-related migraine, Neurosteroid, Neuroactive steroid, Cognitive function, Headache, Pain

## Abstract

**Background:**

Neurosteroids affect the balance between neuroexcitation and neuroinhibition but have been little studied in migraine. We compared the serum levels of pregnenolone sulfate, pregnanolone and estradiol in women with menstrually-related migraine and controls and analysed if a correlation existed between the levels of the three hormones and history of migraine and age.

**Methods:**

Thirty women (mean age ± SD: 33.5 ± 7.1) with menstrually-related migraine (MM group) and 30 aged- matched controls (mean age ± SD: 30.9 ± 7.9) participated in the exploratory study. Pregnenolone sulfate and pregnanolone serum levels were analysed by liquid chromatography-tandem mass spectrometry, while estradiol levels by enzyme-linked immunosorbent assay.

**Results:**

Serum levels of pregnenolone sulfate and pregnanolone were significantly lower in the MM group than in controls (pregnenolone sulfate: *P* = 0.0328; pregnanolone: *P* = 0.0271, Student’s t-test), while estradiol levels were similar. In MM group, pregnenolone sulfate serum levels were negatively correlated with history of migraine (R^2^ = 0.1369; *P* = 0.0482) and age (R^2^ = 0.2826, *P* = 0.0025) while pregnenolone sulfate levels were not age-related in the control group (R^2^ = 0.04436, *P* = 0.4337, linear regression analysis).

**Conclusion:**

Low levels of both pregnanolone, a positive allosteric modulator of the GABAA receptor, and pregnenolone sulfate, a positive allosteric modulator of the NMDA receptor, involved in memory and learning, could contribute either to headache pain or the cognitive dysfunctions reported in migraine patients. Overall, our results agree with the hypothesis that migraine is a disorder associated with a loss of neurohormonal integrity, thus supporting the therapeutic potential of restoring low neurosteroid levels in migraine treatment.

**Supplementary Information:**

The online version contains supplementary material available at 10.1186/s10194-021-01231-9.

## Introduction

Altered levels of neurosteroids, by changing the balance between neuroexcitation and neuroinhibition in the central nervous system (CNS), can affect psychiatric and neurological disorders [1]. However, the involvement of neurosteroids in migraine has been little explored. In a previous study, we found lower levels of allopregnanolone, a positive allosteric modulator of the γ-aminobutyric acid type A (GABAA) receptor, in women with menstrually-related migraine than in controls. These data suggested that the reduced levels of allopregnanolone, an inhibitory neurosteroid, were insufficient to counteract the neuronal hyperexcitability associated with migraine [2], thus contributing to the severity of menstrual related migraine attacks [3]. Indeed, migraine is characterized by generalized neuronal hyperexcitability [4], probably mediated by an increased or impaired glutamate transmission [5]. Therefore, it would also be important to study neuroactive steroids with excitatory functions such as pregnenolone sulfate (pregn-5-en-3β-ol-20-one 3β-sulfate). Actually, this neurosteroid acts as a positive allosteric N-methyl-D-aspartate (NMDA) receptor modulator [6, 7] and is a potent stimulator of transient receptor potential melastatin-3 (TRPM3) channels, that function as nociceptors in the somatosensory system [8]. Moreover, at the molecular level, stimulation of TRPM3 channels has been shown to be involved in calcitonin gene-related peptide (CGRP) exocytosis [9, 10].

Fluctuations in estrogen and progesterone during the oestrus cycle affect pain perception and threshold. Estradiol, by increasing the NMDA receptor activity, is excitatory while progesterone is inhibitory [11]. Furthermore, variation of estrogen levels modulate the signalling of the CGRP receptor in the trigeminovascular system [12, 13]. In the previous study, we found no differences in progesterone levels between women with menstrually-related migraine and non-headache women, but we did not measure estradiol or pregnanolone, an isomer of allopregnanolone, also an agonist of the GABAA receptor [3].

To broaden the study on neurosteroids in menstrually related migraine and, in particular, to investigate the balance between steroids with inhibitory and excitatory functions we determined the serum levels of pregnenolone sulfate, pregnanolone and estradiol in women suffering from menstrually-related migraine, compared them with those in non-headache women and analysed whether the levels of the three hormones correlated with the history of migraine and age.

## Methods

### Study population

We determined the serum concentrations of pregnenolone sulfate, estradiol and pregnanolone in the same samples in which we had previously determined other neuroactive hormones (allopregnanolone, progesterone and testosterone) [3]. Therefore, this exploratory study had involved 30 women (mean age ± SD: 33.5 ± 7.1; range 19–45; mean years of migraine ± SD: 17.4 ± 8.9 years) diagnosed with menstrually-related migraine according to the diagnostic criteria of the International Classification of Headache Disorders 3rd edition (ICHD-3, appendix, A1.1.2) [14] (MM group) and 30 non-headache, age-matched women, as controls (mean age ± SD: 30.9 ± 7.9; range 18–44).

Only women with no medical or psychiatric comorbidities and who were not taking migraine prophylaxis, hormone therapies or other drugs capable of modifying neurosteroid levels were included in the study. The MM group was enrolled among the patients of the Headache Center of the University Hospital of Modena (Italy); controls were acquaintances of the patients. The subjects were enrolled from July 2018 to May 2019. All the women provided written consent to participate in the study which was approved by the Ethics Committee of the Area Vasta Emilia Nord (AVEN, Italy) (prot. 0013510/18). Study design and enrollment’s procedures are described in detail in our previous publication [3].

### Data collection and procedure

For each subject, personal data, lifestyle habits, clinical history and, for the migraine group, also the history of migraine were collected. Fasting blood samples were collected between day 7 and day 10 of the menstrual cycle and at least two days after the last migraine attack. The samples were allowed to clot at room temperature for 1 h and then centrifuged at 2000 x g for 10 min at + 4 °C to collect sera.

### Quantitative determination of pregnenolone sulfate and pregnanolone by liquid chromatography-tandem mass spectrometry (LC-MS/MS)

Quantitative determination of pregnenolone sulfate and pregnanolone, which present a keto-group in their moieties, was performed as previously described [3]. Briefly, serum samples were added with deuterated internal standard (IS), vortexed and treated with acetonitrile/methanol (70/30; + 1.0% formic acid) to precipitate proteins. The obtained supernatants were subjected to solid phase extraction procedure to remove endogenous phospholipids and evaporated to dryness. The residues were then derivatized with Amplifex Keto Reagent and analysed by LC-MS/MS on a Kinetex XB-C18 column with a mobile phase of water/acetonitrile (+ 3 mM ammonium formate, + 0.1% formic acid) under gradient elution. Compounds were detected in multiple reaction monitoring mode by acquiring three selected MS/MS transitions for each analyte and IS. Serum levels of the target analytes were determined via the calibration curves calculated by analysing blank albumin samples, spiked with known amounts of the target analytes and IS and purified as described above (x = concentration; y = analyte signal/IS signal ratio). Calibration range was 4.00–120 ng/ml and 0.02–0.65 ng/ml for pregnenolone sulfate and pregnanolone, respectively, and limit of quantitation values were 0.010 ng/mL and 0.006 ng/mL for pregnenolone sulfate and pregnanolone, respectively.

### Quantitative determination of estradiol by enzyme-linked Immunosorbent assay (ELISA)

Estradiol, whose molecule lacks a carbonyl group, does not respond to Amplifex derivatization. On the other hand, direct LC-MS/MS analysis of non-derivatized estradiol does not provide sufficient sensitivity to detect low concentrations. Consequently, the enzyme immunoassay technique was employed for the quantitative measurement of estradiol in serum using a commercial kit (Estradiol sensitive ELISA, Demeditec, Germany), following the protocol reported by the manufacturer. The data were acquired on a microplate reader (Multiscan FC, Thermo Scientific, USA) by measuring the absorbance at λ = 450 nm. Estradiol concentrations were determined from a standard curve generated with calibrators included in the kit (calibration range: 1.4–200 pg/mL; limit of quantitation: 1.4 pg/mL).

### Statistical analysis

Serum levels of pregnenolone sulfate, pregnanolone and estradiol determined in the MM group were compared with those found in the control group by Student’s t-test for independent samples. Effect sizes were calculated using Cohen’s d to compare magnitude of the difference of the means and considered as small (0.2), medium (0.5) and large (0.8). The relationship between the levels of the three hormones (independent variables) with migraine years and age was assessed by linear regression analysis. Statistical analysis was carried out by StataIC 13 software. Differences were considered significant if *P*-value (two-tailed) was lower than 0.05.

## Results

In the MM group, both pregnenolone sulfate (39.58 ± 18.36) and pregnanolone (0.09 ± 0.06) serum levels (ng/mL, mean ± SD) were significantly lower than in the control group (pregnenolone sulfate: 55.82 ± 31.79, *P* = 0.0328; pregnanolone: 0.16 ± 0.11, *P* = 0.0271, Student’s t-test). The size of the differences between MM and controls determined by Cohen’s d value was medium for pregnenolone sulfate (− 0.64) and large for pregnanolone (− 0.82). Serum levels (pg/mL, mean ± SD) of estradiol were similar between MM (90.02 ± 59.22) and control (67.52 ± 41.12) groups (*P* = 0.1538, Student’s t-test).

**Fig. 1:**
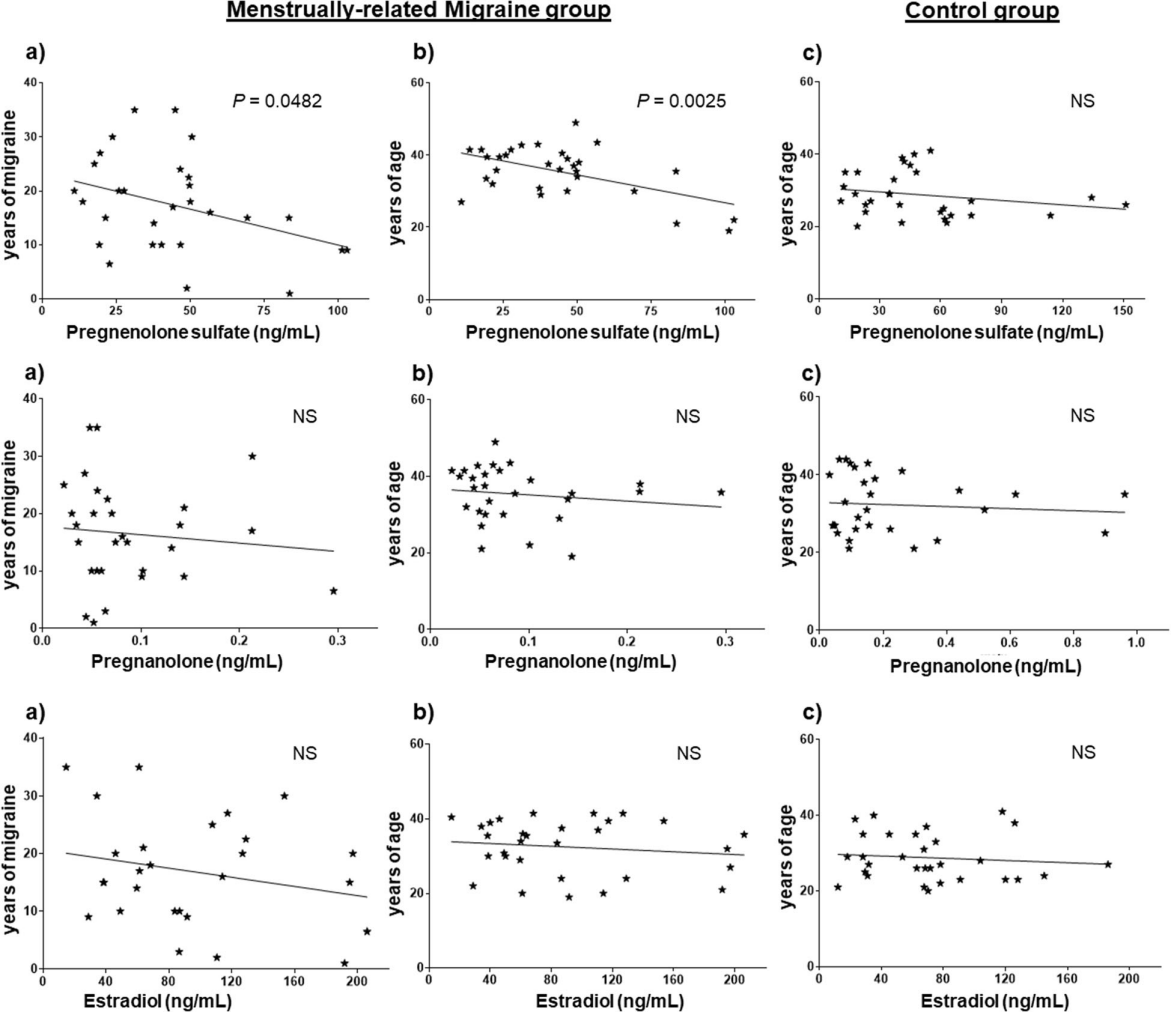
Correlation between serum levels of pregnenolone sulfate, pregnanolone and estradiol and **a**) years of migraine in MM group, **b**) years of age in MM group and **c**) years of age in control group. NS = not significant

In the MM group (Fig. 1) pregnenolone sulfate serum levels were negatively correlated with history of migraine (R^2^ = 0.1369; *P* = 0.0482) and age (R^2^ = 0.2826, *P* = 0.0025) while pregnenolone sulfate levels were not age-related in the control group (R^2^ = 0.04436, *P* = 0.4337, linear regression analysis). The levels of pregnanolone and estradiol in the MM group showed no correlation with history of migraine (pregnanolone: R^2^ = 0.01091, *P* = 0.5897; estradiol: R^2^ = 0.04694, *P* = 0.2777) and age (pregnanolone: R^2^ = 0.02108, *P* = 0.4524; estradiol: R^2^ = 0.02805, *P* = 0.4037). Even in the control group, pregnanolone and estradiol levels showed no correlation with age (pregnanolone: R^2^ = 0.01182, *P* = 0.6997; estradiol: R^2^ = 0.001884, *P* = 0.8779, linear regression analysis).

## Discussion

Neuroactive steroids modulate a broad spectrum of neuronal functions including cognition, memory and pain perception. In particular, pregnenolone sulfate acts as a cognitive enhancer by improving learning and memory [1, 15] and reduced pregnenolone sulfate levels could be involved in the cognitive dysfunction and memory problems reported in migraine patients [16]. Accordingly, in MM women the serum levels of pregnenolone sulfate (Fig. 1) correlated inversely with the history of migraine and age, while in the control group no correlation with age was present. Therefore, we believe that migraine and its persistence over the years, rather than the age alone, were associated with progressively decreased levels of pregnenolone sulfate.

The levels of pregnanolone were also significantly lower in MM compared to the control group, in accordance with the previously detected low concentrations of allopregnanolone [3], but they did not correlate with the history of migraine or age. This data can be explained bearing in mind that the production routes of the two hormones are different [17].

As we did not find any differences for progesterone in the previous study [3], the levels of estradiol in the follicular phase were similar between MM and control groups and did not correlate with the history of migraine or age. In contrast, estradiol levels in the luteal phase were reported to be significantly lower in patients suffering from menstrually-related migraine than in healthy controls [18].

Our study has some limitations. The main one was that the determinations were performed in peripheral blood and the ability of pregnenolone sulfate to penetrate the blood brain barrier (BBB) is questionable. It could simply contribute to the neuroactive steroid pool or penetrate the BBB via transporter proteins [19]. However, circulating pregnenolone sulfate has been observed to affect pregnenolone concentration in the CNS in humans [17]. Finally, peripheral levels of allopregnanolone and pregnenolone have been shown to be positively correlated with gray matter thickness in multiple regions of the cerebral cortex [20]. Ours was an exploratory study, of limited number and therefore our results need to be confirmed in a wider series. We did not perform cognitive tests in the two studied groups, which however did not differ in demographic characteristics and comorbidities. In addition, the levels of pregnenolone sulfate in the control group agreed with those reported in the literature for healthy subjects [1, 21], while no other published data are available for levels in migraine.

Overall, the current results concerning lower levels for pregnenolone sulfate and pregnanolone and our previous results for lower allopregnanolone serum levels in MM group versus control group (see Additional Table) [3] agree with the hypothesis that migraine is a disorder associated with a loss of neurohormonal integrity [22]. In fact, it has been observed that severe pain causes hyper-excitation of the hypothalamus-pituitary-adrenal system which results in high levels of serum hormones such as adrenocorticotropin, cortisol and pregnenolone. If the severe pain continues, however, the system cannot maintain its normal hormone production and the serum levels of some hormones can drop below the normal range [23].

**Fig. 2:**
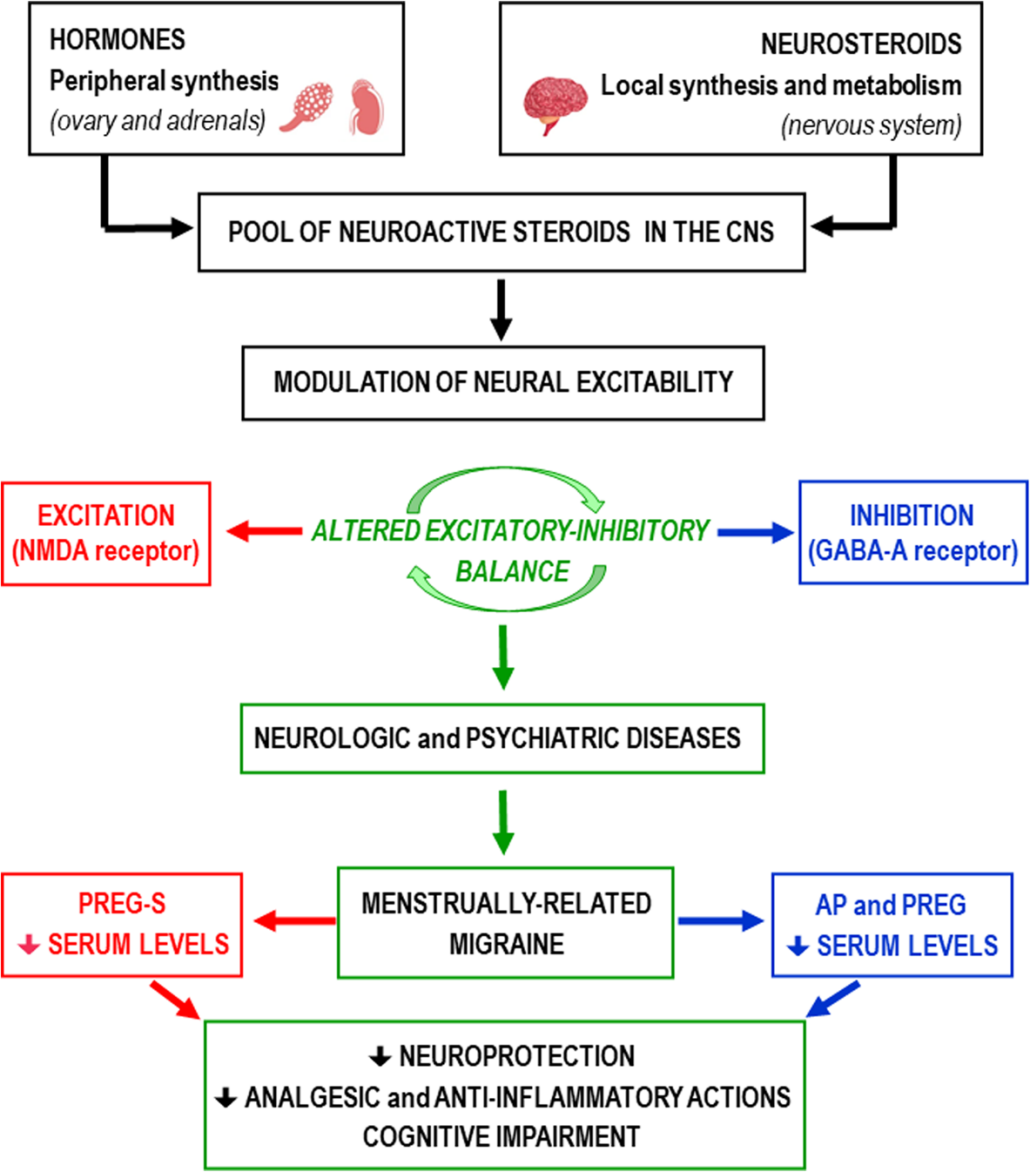
Neurosteroids in menstrually-related migraine (MM): altered serum levels in the analyzed samples and possible pathophysiological role. Serum levels of estradiol, progesterone and testosterone were similar between MM and control groups in our studies (samples collected in follicular phase). CNS: central nervous system; PREG-S: pregnenolone sulfate; AP: allopregnanolone; PREG: pregnanolone. Image credit: https://www.shutterstock.com

In conclusion, the reduction in peripheral levels of neuroactive steroids in women with menstrually-related migraine (Fig. 2), influencing the neurosteroid pool in the CNS and the activity of neuronal networks, could contribute either to headache pain or the cognitive dysfunctions reported in migraine [16, 24, 25]. Consequently, restoring low endogenous neurosteroid levels could exhibit therapeutic potential in migraine management.

## Supplementary Information

**Additional file 1.**

## Data Availability

Anonymized data operated or analysed during this study are available from the Authors upon reasonable request.

## Abbreviations

MM: Menstrually-related migraine
CNS: Central nervous system
GABAA: γ-aminobutyric acid type A
NMDA: N-methyl-D-aspartate
TRPM3: Transient receptor potential melastatin-3
CGRP: Calcitonin gene-related peptide
ICHD-3: International Classification of Headache Disorders 3rd edition
LC-MS/MS: Liquid chromatography-tandem mass spectrometry
IS: Internal standard
ELISA: Enzyme-Linked Immunosorbent Assay
BBB: Blood brain barrier

## References

[1] Ratner MH, Kumaresan V, Farb DH (2019). Neurosteroid actions in memory and neurologic/neuropsychiatric Disorders. Front Endocrinol (Lausanne).

[2] Younis S, Christensen CE, Vestergaard MB, Lindberg U, Tolnai D, Paulson OB, et al (2021) Glutamate levels and perfusion in pons during migraine attacks: a 3T MRI study using proton spectroscopy and arterial spin labeling. J Cereb Blood Flow Metab. 41(3):604-616. 10.1177/0271678X20906902. 10.1177/0271678X20906902 PMC7922760 32423331

[3] Rustichelli C, Bellei E, Bergamini S, Monari E, Baraldi C, Castro FL, Tomasi A, Ferrari A (2020). Serum levels of allopregnanolone, progesterone and testosterone in menstrually-related and postmenopausal migraine: a cross-sectional study. Cephalalgia..

[4] Burstein R, Noseda R, Borsook D (2015). Migraine: multiple processes, complex pathophysiology. J Neurosci.

[5] Hoffmann J, Charles A (2018). Glutamate and its receptors as therapeutic targets for migraine. Neurotherapeutics..

[6] Chisari M, Wilding TJ, Brunwasser S, Krishnan K, Qian M, Benz A, Huettner JE, Zorumski CF, Covey DF, Mennerick S (2019). Visualizing pregnenolone sulfate-like modulators of NMDA receptor function reveals intracellular and plasma-membrane localization. Neuropharmacology..

[7] Hrcka Krausova B, Kysilov B, Cerny J, Vyklicky V, Smejkalova T, Ladislav M, Balik A, Korinek M, Chodounska H, Kudova E, Vyklicky L (2020). Site of action of brain neurosteroid pregnenolone sulfate at the N-methyl-D-aspartate receptor. J Neurosci.

[8] Méndez-Reséndiz KA, Enciso-Pablo Ó, González-Ramírez R, Juárez-Contreras R, Rosenbaum T, Morales-Lázaro SL (2020). Steroids and TRP channels: a close relationship. Int J Mol Sci.

[9] Thiel G, Rubil S, Lesch A, Guethlein LA, Rössler OG (2017). Transient receptor potential TRPM3 channels: pharmacology, signaling, and biological functions. Pharmacol Res.

[10] Benemei S, Dussor G (2019). TRP channels and migraine: recent developments and new therapeutic opportunities. Pharmaceuticals.

[11] Finocchi C, Strada L (2014). Sex-related differences in migraine. Neurol Sci.

[12] Labastida-Ramírez A, Rubio-Beltrán E, Villalón CM, MaassenVanDenBrink A (2019). Gender aspects of CGRP in migraine. Cephalalgia..

[13] Cupini LM, Corbelli I, Sarchelli P (2020) Menstrual migraine: what it is and does it matter? J Neurol. 10.1007/s00415-020-09726-2 10.1007/s00415-020-09726-2 31989282

[14] (2018) Headache classification Committee of the International Headache Society (IHS) the international classification of headache disorders, 3rd edition. Cephalalgia. 38(1):1–211. 10.1177/0333102417738202 10.1177/0333102417738202 29368949

[15] Belelli D, Hogenkamp D, Gee KW, Lambert JJ (2020). Realising the therapeutic potential of neuroactive steroid modulators of the GABAA receptor. Neurobiol Stress.

[16] Vuralli D, Ayata C, Bolay H (2018) Cognitive dysfunction and migraine. J Headache Pain 19(1):109. 10.1186/s10194-018-0933-4. 10.1186/s10194-018-0933-4 PMC6755588 30442090

[17] Kancheva R, Hill M, Novák Z, Chrastina J, Kancheva L, Stárka L (2011). Neuroactive steroids in periphery and cerebrospinal fluid. Neuroscience..

[18] Ibrahimi K, van Oosterhout WPJ, van Dorp W, Danser AHJ, Garrelds IM, Kushner SA, Lesaffre EMEH, Terwindt GM, Ferrari MD, van den Meiracker AH, MaassenVanDenBrink A (2015). Reduced trigeminovascular cyclicity in patients with menstrually related migraine. Neurology..

[19] Grube M, Hagen P, Jedlitschky G (2018). Neurosteroid transport in the brain: role of ABC and SLC transporters. Front Pharmacol.

[20] Morey RA, Davis SL, Haswell CC, Naylor JC, Kilts JD, Szabo ST, Shampine LJ, Parke GJ, Sun D, Swanson CA, Wagner HR, Marx CE, Mid-Atlantic MIRECC Workgroup (2019). Widespread cortical thickness is associated with neuroactive steroid levels. Front Neurosci.

[21] Heydari B, Le Mellédo J-M (2002). Low pregnenolone sulphate plasma concentrations in patients with generalized social phobia. Psychol Med.

[22] Dzugan SA, Dzugan KS (2015). Is migraine a consequence of a loss of neurohormonal and metabolic integrity? A new hypothesis. Neuro Endocrinol Lett.

[23] Tennant F (2013). The physiologic effects of pain on the endocrine system. Pain Ther.

[24] Santangelo G, Russo A, Tessitore A, Garramone F, Silvestro M, Della Mura MR, Marcuccio L, Fornaro I, Trojano L, Tedeschi G (2018). Prospective memory is dysfunctional in migraine without aura. Cephalalgia..

[25] Gil-Gouveia R, Martins IP (2019). Cognition and cognitive impairment in migraine. Curr Pain Headache Rep.

