# Supplementary material for: Comparison of pregnenolone sulfate, pregnanolone and estradiol levels between patients with menstrually-related migraine and controls: an exploratory study
Source: J Headache Pain. 2021 Mar 23;22(1):13. doi: 10.1186/s10194-021-01231-9 (PMC7989028; doi:10.1186/s10194-021-01231-9)
Supplement: Supplementary file 1 — Additional file 1. [file 10194_2021_1231_MOESM1_ESM.docx]

**Additional Table** Serum levels of the analyzed neurosteroids and related negative correlations in menstrually-related migraine (MM) and control groups from current and previous study [3].

| **Variable** | **MM group** | **Control group** |
| --- | --- | --- |
|  | *Pregnenolone sulfate* | |
| Serum levels  (ng/mL, mean ± SD) | 39.58 ± 18.36 | 55.82 ± 31.79  *P* = 0.0328 |
| Correlation with years of migraine | R^2^ = 0.1369  *P* = 0.0482 |  |
| Correlation with age (years) | R^2^ = 0.2826  *P* = 0.0025 | R^2^ = 0.04436  *P* = 0.4337 |
|  | *Pregnanolone* | |
| Serum levels  (ng/mL, mean ± SD) | 0.09 ± 0.06 | 0.16 ± 0.11  *P* = 0.0271 |
| Correlation with years of migraine | R^2^ = 0.01091  *P* = 0.5897 |  |
| Correlation with age (years) | R^2^ = 0.02108  *P* = 0.4524 | R^2^ = 0.01182  *P* = 0.6997 |
|  | *Estradiol* | |
| Serum levels  (pg/mL, mean ± SD) | 90.02 ± 59.22 | 67.52 ± 41.12  *P* = 0.1538 |
| Correlation with years of migraine | R^2^ = 0.04694  *P* = 0.2777 |  |
| Correlation with age (years) | R^2^ = 0.02805  *P* = 0.4037 | R^2^ = 0.001884  *P* = 0.8779 |
| **Previous data [3]** | | |
|  | *Allopregnanolone* | |
| Serum levels  (pg/mL, mean ± SD) | 52 ± 18 | 78 ± 36  *P* < 0.001 |
| Correlation with years of migraine | R^2^ = 0.13  *P* = 0.069 |  |
|  | *Progesterone* | |
| Serum levels  (pg/mL, mean ± SD) | 135 ± 47 | 162 ± 120  *P* = 0.30 |
|  | *Testosterone* | |
| Serum levels  (pg/mL, mean ± SD) | 298 ± 116 | 327 ± 124  *P* = 0.43 |
